# Bone remodeling and implant migration of uncemented femoral and cemented asymmetrical tibial components in total knee arthroplasty - DXA and RSA evaluation with 2-year follow up

**DOI:** 10.1186/s43019-021-00111-5

**Published:** 2021-08-17

**Authors:** Müjgan Yilmaz, Christina Enciso Holm, Thomas Lind, Gunnar Flivik, Anders Odgaard, Michael Mørk Petersen

**Affiliations:** 1 Department of Orthopedic Surgery, University Hospital of Copenhagen, Rigshospitalet, Inge Lehmanns Vej 6, 2100 Copenhagen Ø, Denmark; 2 Department of Orthopedic Surgery, University Hospital of Copenhagen, Herlev-Gentofte Hospital, Gentofte Hospitalsvej 1, 2900 Hellerup, Denmark; 3 Department of Clinical Medicine, Faculty of Health and Medical Sciences, University of Copenhagen, Copenhagen, Denmark; 4 Department of Orthopedics, Skane University Hospital, Clinical Sciences, Lund University, Entrégaten 7, 222 42 Lund, Sweden

**Keywords:** Total knee replacement, Total knee arthroplasty, Persona®, MBRSA, DXA

## Abstract

**Background:**

Aseptic loosening is one of the major reasons for late revision in total knee arthroplasty (TKA). The risk of aseptic loosening can be detected using radiostereometric analysis (RSA), whereby micromovements (migration) can be measured, and thus RSA is recommended in the phased introduction of orthopedic implants. Decrease in bone mineral density (BMD), as measured by dual-energy x ray absorptiometry (DXA), is related to the breaking strength of the bone, which is measured concurrently by RSA. The aim of the study was to evaluate bone remodeling and implant migration with cemented asymmetrical tibial and uncemented femoral components after TKA with a follow up period of 2 years.

**Methods:**

This was a prospective longitudinal cohort study of 29 patients (number of female/male patients 17/12, mean age 65.2 years), received a hybrid Persona® TKA (Zimmer Biomet, Warsaw, IN, USA) consisting of a cemented tibial, an all-polyethylene patella, and uncemented trabecular metal femoral components. Follow up: preoperative, 1 week, and 3, 6, 12 and 24 months after surgery, and double examinations for RSA and DXA were performed at 12 months. RSA results were presented as maximal total point of motion (MTPM) and segmental motion (translation and rotation), and DXA results were presented as changes in BMD in different regions of interest (ROI).

**Results:**

MTPM at 3, 6, 12, and 24 months was 0.65 mm, 0.84 mm, 0.92 mm, and 0.96 mm for the femoral component and 0.54 mm, 0.60 mm, 0.64 mm, and 0.68 mm, respectively, for the tibial component. The highest MTPM occurred within the first 3 months. Afterwards most of the curves flattened and stabilized. Between 12 and 24 months after surgery, 16% of femoral components had migrated by more than 0.10 mm and 15% of tibial components had migrated by more than 0.2 mm. Percentage change in BMD in each ROI for distal femur was as follows: ROI I 26.7%, ROI II 9.2% and ROI III 3.3%. BMD and at the proximal tibia: ROI I 8.2%, ROI II 8.6% and ROI III 7.0% after 2 years compared with 1 week postoperative results. There was no significant correlation between maximal percentwise change in BMD and MTPM after 2 years.

**Conclusion:**

Migration patterns and changes in BMD related to femoral components after TKA in our study correspond well with previous studies; we observed marginally greater migration with the tibial component.

**Supplementary Information:**

The online version contains supplementary material available at 10.1186/s43019-021-00111-5.

## Introduction

Total knee arthroplasty (TKA) is, in general, a very successful treatment for patients with symptomatic osteoarthritis (OA), and register studies indicate implant survival of more than 90% after 10 years [1, 2]. One of the major causes of long-term revision is aseptic loosening [1, 3].

The risk of aseptic loosening can be detected by radiostereometric analysis (RSA), whereby micromovements, described as migration, can be measured, and thus RSA is recommended as a standard in the phased introduction of new orthopedic implants [4] with 2-year follow up [5]. Migration is seen with both cemented and uncemented implants but most implants stabilize during the first postoperative year; however, some implants migrate continuously, and this incurs high risk of subsequent aseptic loosening and implant revision [6, 7]. With tantalum markers attached to the polyethylene insert and bone, small micromovements of the implant can be detected using marker-based RSA [8]. Model-based RSA (MBRSA), used in this study, has been developed from marker-based RSA; the precision error of this technique has been found to be acceptable and does not require tantalum markers attached to the polyethylene insert [9, 10]. RSA is highly accurate and can be performed in small study populations [6].

Dual-energy x ray absorptiometry (DXA) can be used to measure changes in bone mineral density (BMD) after TKA [11, 12]; a significant decrease in BMD is often seen after TKA in both the proximal tibia [13–17] and the distal femur [18]. Since BMD "is strongly related to the breaking strength of bone" [19–21], at least for theoretical reasons, we believe that change in BMD where an implant is anchored is another important and relevant parameter in the early phase when introducing a new implant for clinical use, and maybe correlation between migration and BMD can be detected.

The aim of this study was to evaluate implant migration using MBRSA and bone remodeling using DXA, and to assess correlation between implant migration and bone remodeling in patients with cemented asymmetrical tibial and uncemented femoral TKA components over a follow up period of 2 years.

## Material and methods

### Patients

**Fig. 1:**
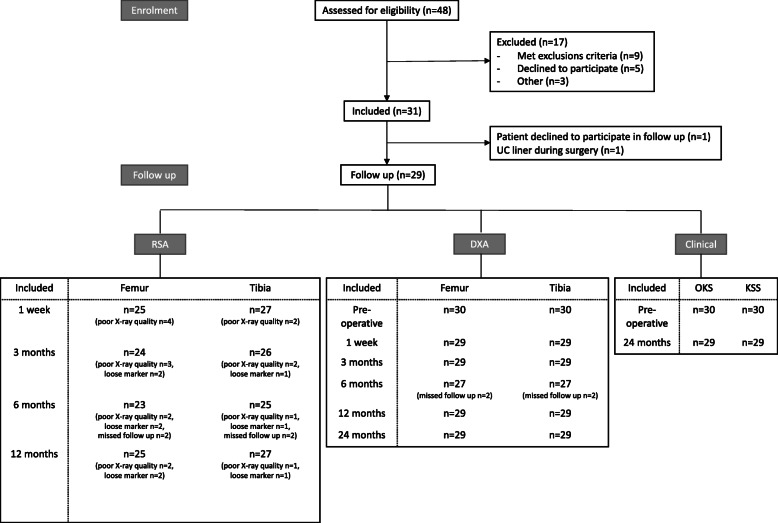
Enrolment overview. Twenty-nine patients were included in the follow up. Two patients did not attend follow up at 6 months. One patient did not undergo preoperative dual-energy x ray absorptiometry (DXA) and clinical assessment. RSA, radiostereometric analysis; UC, ultra-congruent

We performed a prospective longitudinal cohort study of patients (Fig. 1) (demographics are shown in Table 1) who underwent primary hybrid TKA for treatment of OA at Gentofte Hospital between 21 March and 12 October 2017. Patients between the ages of 40 and 70 years, diagnosed with OA and scheduled for primary TKA were included in the study after providing informed consent. Patients with diseases that could influence bone metabolism, patients who did not comprehend the given information, and patients who declined to participate were excluded. The hybrid Persona® (Zimmer Biomet, Warsaw, IN, USA) TKA implant consists of a cemented asymmetrical tibial, uncemented trabecular metal (TM) femoral, cruciate-retaining (CR) polyethylene insert and cemented all-polyethylene patella components. All surgery was performed by three experienced knee surgeons following guidelines provided by the manufacturer.

**Table 1:** Demographic overview

	All (***n*** = 29)	Female (***n*** = 17)	Male (***n*** = 12)
**Mean age at surgery in years (range)**	65.1 (52.8–70)	63.8 (52.8–70)	67.1 (53.3–69.7)
**Weight in kg (range)**	85.4 (58–120)	81.6 (58–114)	92 (75–120)
**BMI (range)**	29.2 (18.5–41.5)	29.1 (18.5–38)	30 (23.2–41.5)
**Smoking**			
Never:	15	9	6
Current:	4	2	2
Former:	10	6	4
**Anesthesia**			
General:	10	6	4
Spinal:	19	11	8
**Polyethylene inserts in mean mm (range)**	12 (10–16)	12 (10–14)	12 (10–16)
**Patella size**			
32:	7	6	1
35:	17	11	6
38:	5	–	5
**Femur component size**			
5:	2	1	1
6:	3	3	–
7:	5	4	1
8:	6	4	2
9:	6	5	1
10:	2	–	2
11:	5	–	5
*25 Standard and 4 narrow components			
**Tibia component size**			
D:	5	5	–
E:	8	7	1
F:	7	5	2
G:	7	–	7
H:	2	–	2

We included 31 patients in the study; 29 patients were available for follow up as 1 patient declined to participate in the study after surgery, 1 patient had a change of tibial insert to an ultra-congruent (UC) during initial surgery, and 1 patient did not attend to the preoperative appointment (but are still included due to 1 week RSA are used as a baseline for further analysis) (Fig. 1). No revision surgery was performed.

### RSA

During surgery, at least six tantalum beads (0.8 mm, Tilly Medical Products, Lund, Sweden) were placed in both the proximal tibia, the polyethylene insert and the distal femur, using an inserter that positions and inserts markers in bone one at a time (Wennbergs Finmark AB, Gunnilse, Sweden). The same assistant positioned the beads in each procedure to minimize variation and we aimed for the widest possible non-linear spread between the beads. The tantalum markers placed in the polyethylene insert were not used for the analyses in this study because MBRSA was used to evaluate migration and segmental motion.

RSA performed 1 week after surgery (mean 7.8, range 6–13 days) was used as the baseline for RSA measurements and follow up examinations were performed at 3, 6, 12, and 24 months after surgery. RSA was performed with the patient in a standardized supine position, with the knee placed in a biplane plexiglass calibration cage (Calibration cage 21; Tilly Medical Products, Lund, Sweden). Two moveable ceiling-fixed x ray tubes (Arcoma Precision T3, Siemens, 0.7mm AI/75 kV, filtration 1.5 mm) were positioned at a 90° angle to each other, one positioned for the anterior-posterior projection and the other for the medial-lateral projection. Both tubes were placed 100 cm from the x ray detectors in moveable cassettes, and intensity was set at 50 kV and 25 mA seconds (mAs). The radiographic images were stored in digital imaging and communication in medicine (DICOM) format with a resolution of 10 pixels per millimeter, in the picture archiving and communication system (PACS). All examinations were performed by the same two researchers.

**Fig. 2:**
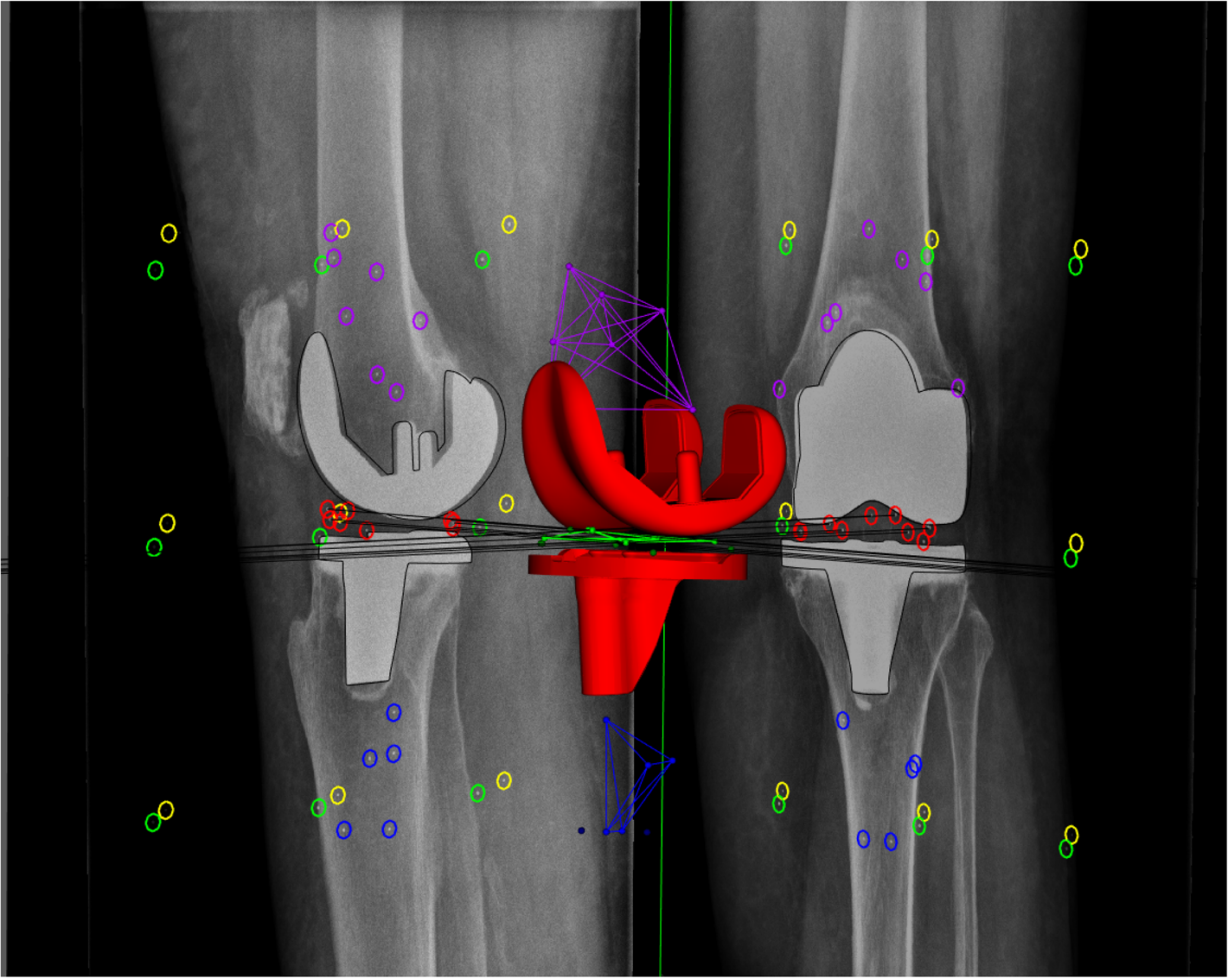
Radiostereometric analysis (RSA) images with computer-aided design (CAD) model during analyses

RSA analysis (Fig. 2) was performed using model-based software [22, 23] (Model-based RSA 4.1, 2003–2014 RSAcore Department of orthopedics Leiden University Medical Center) in cooperation with the department of orthopedics, Skane University Hospital in Lund, Sweden. Computer-aided design (CAD) [23] models were delivered from Leiden (RSAcore Department of orthopedics Leiden University Medical Center) based on prosthesis design information from the company.

The distribution of tantalum markers is expressed by the condition number (CN), whereas mean error (ME) is an expression of the stability of the tantalum markers; both CN and ME are calculated by the analysis software. We were aiming for a low CN, which indicates a non-linear distribution with wide dissemination of the markers. The upper limits for CN and ME were set at 150 and 0.35 mm, respectively, according to guidelines [24]. Migration is presented as maximal total point of motion (MTPM), which represents the point of maximum motion and is highly sensitive for loose markers (tantalum beads attached to the bone). Segmental motion is expressed as translation along the X (medial-lateral), Y (proximal-distal) and Z (anterior-posterior) axes and rotation X (flexion-extension), Y (internal-external) and Z (valgus-varus).

Double RSA radiographic images (*n* = 22) were obtained at the 12-month follow up. Patients were requested to stand up between each examination and were positioned again after 5 min in the aforementioned supine position and additional RSA radiographic images were obtained. We evaluated the measurement precision for RSA. Precision was defined as the standard deviation of the difference (SD_diff_) and precision error was expressed as 1.96 x SD_diff_ [24].

### DXA

DXA was performed 1 week postoperatively (mean 7.8, range 6–13 days), and after 3, 6, 12, and 24 months. The distal femur in the affected limb was scanned in the sagittal plane, with the patient positioned in the lateral decubitus position, with the affected knee placed nearest to the examination table and in slight flexion, to obtain a true lateral projection. The proximal tibia on the affected limb was scanned in the anterior-posterior plane, with the patient placed in the supine position with the knee fully extended and the lower limb slightly rotated inward, to avoid superimposition of the fibula and tibia.

DXA was performed by two experienced technicians using a Norland XR-46 bone densitometer (Norland Corp., Fort Atkinson, WI, USA). The proximal tibia and distal femur were scanned using customized software for research with a pixel size of 0.5 × 0.5 mm and a speed of 45 mm/sec.

**Fig. 3:**
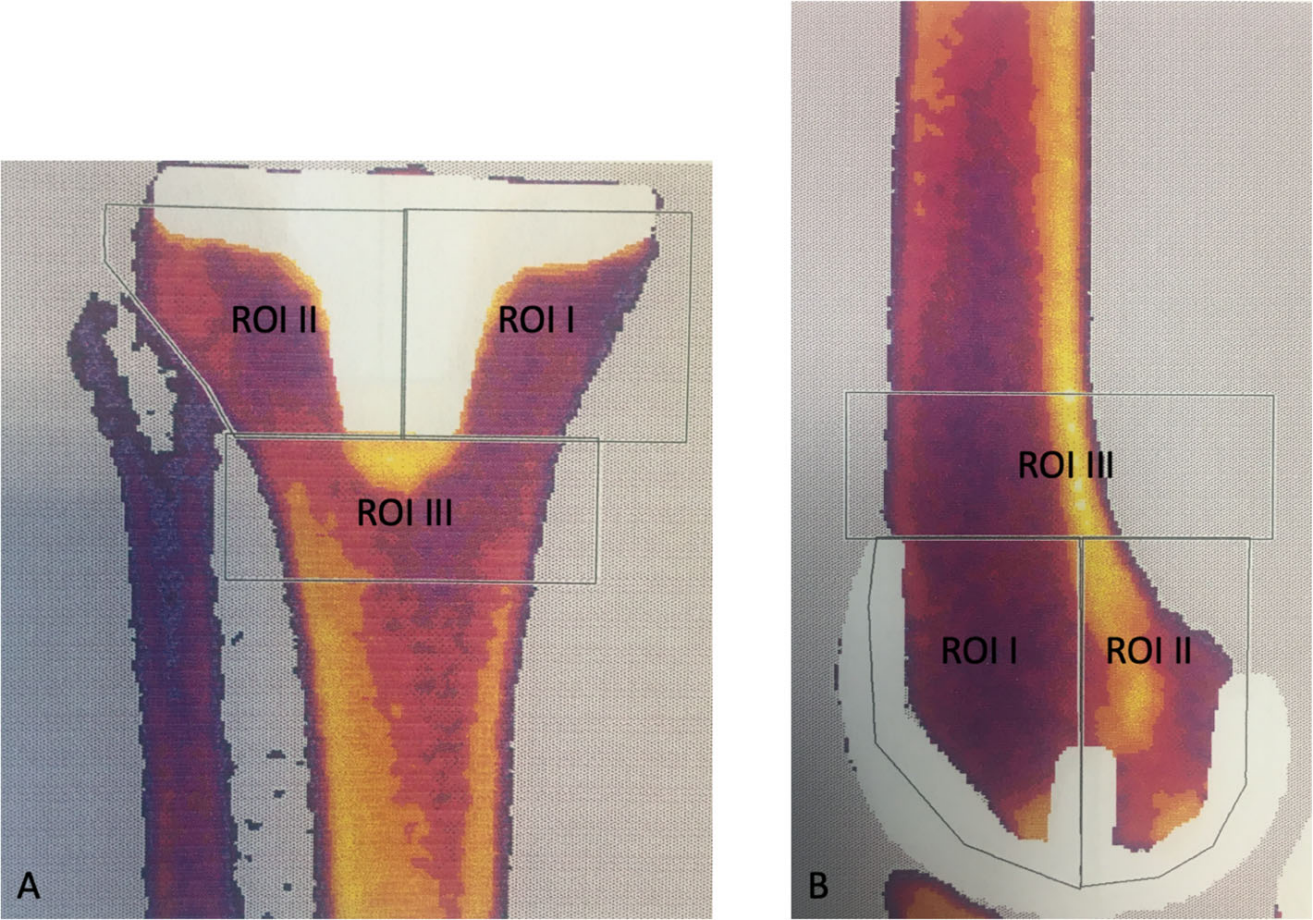
Dual-energy x ray absorptiometry (DXA) scan of proximal tibia illustrating region of interest (ROI) I (medial), ROI II (lateral), and ROI III (distal) (**A**) and of distal femur illustrating ROI I (anterior), ROI II (posterior) and ROI III (proximal) (**B**)

Both femoral and tibial DXA scans were analyzed by creating three regions of interest (ROI) on the computerized scan plots (Fig. 3A and B) for measurement of BMD.

Double DXA scans (*n* = 16) were obtained at the 12-month follow up. Patients were requested to stand up between each examination and were positioned again after 5 min using the aforementioned positioning and then rescanned. The precision error of the BMD measurements in the various ROI of the proximal tibia and the distal femur was calculated from the double measurements and expressed as the mean coefficient of variation (CV) (CV = (standard deviation (SD)/mean) × 100%).

### Clinical follow up

The Knee Society score (KSS) and the Oxford knee score (OKS) were calculated preoperatively and postoperatively after 1 and 2 years. The KSS is a physician-completed score and consists of a clinical and a functional score. Clinical scores include pain, extension lag, total range of flexion, alignment, stability (anterior-posterior and mediolateral), and if present, flexion contracture. Functional scores include quality of walking, whether walking aids are used, and the ability to use stairs. A KSS score below 60 is considered poor, 60–69 fair, 70–79 good, and 80–100 excellent [25].

The OKS is a patient-reported score and consists of 12 items to assess function during the past 4 weeks, where a score of 0 (minimum) may indicate severe OA and 48 (maximum) may indicate satisfactory function [26].

### Statistical analysis and ethical statements

Data on MBRSA translation (millimeters) and rotation (degrees) were expressed as mean values with 95% confidence intervals (95CI). As recommended by Valstar et al. [24], all translation and rotation values were presented as signed values. The *t* test for paired data was used to compare time-related change (0–24 months) in BMD, and percentage time-related mean change in BMD was presented with 95CI. The OKS and KSS were expressed as the mean with 95CI and preoperative and 2-year followup values were compared using the paired *t* test.

The size of our study population size corresponds well with the number of required participants as determined from previous sample size calculations for RSA and DXA studies when comparing two different implants. RSA has high accuracy and therefore a small number of participants can be studied [24].

Mean annual migration of 0.09–0.10 mm for femoral components is comparable with a good long-term outcome [7, 27]. According to Pijls et al. [28], after 1 year, tibial components with a MTPM ≤ 0.54 mm are classified as acceptable, those with MTPM of 0.55–1.6 mm are classified as at risk, and those with MTPM > 1.6 are classified as unacceptable. Revision in 2018 [29] indicates MTPM < 0.5 mm at 6 months is an indicator of good clinical outcome. Annual migration ≤ 0.2 mm indicates stabilization and a good predictable factor [6]. Statistical analyses were executed in RStudio® (Version 1.2.1335© 2009–2019 RStudio, inc.).The level of statistical significance was set at *p* <0.05 and confidence intervals were reported at 95%.

Approval from the local Ethical Committee (case no. H-16035883) and Danish Data Protection Agency (case no. 2012-58-0004, RH-2017-36 and I-Suite nr: 05264) was obtained. All patients were informed about the study orally and in writing by the principal investigator and informed consent was obtained prior to inclusion, in accordance with the Helsinki Declaration.

## Results

### RSA

#### Femur

The precision error for measurement of MTPM from 22 femoral double examinations was 0.19 mm. Precision error for the segmental motion was 0.20°, 0.25°, and 0.24° for X, Y, and Z rotations, respectively, and precision error for the corresponding translational segmental motion was 0.16 mm, 0.07 mm, and 0.18 mm, respectively.

**Fig. 4:**
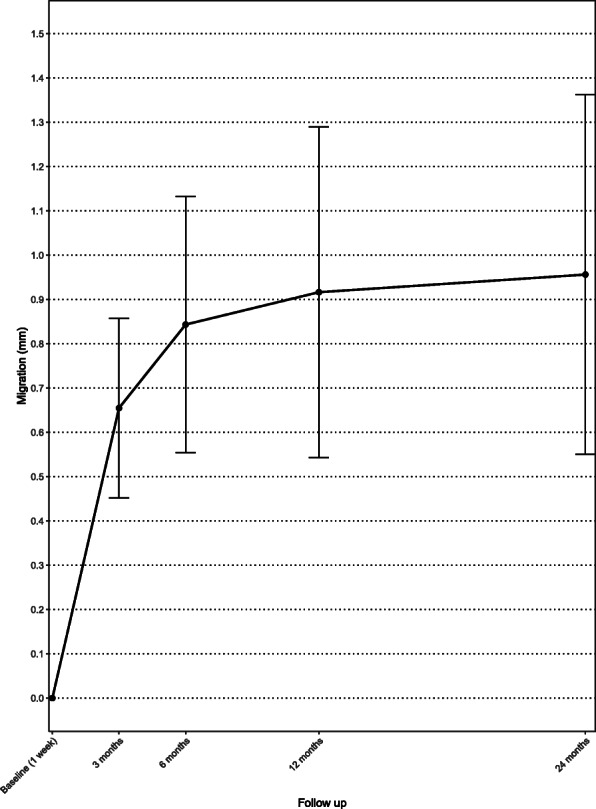
Mean maximal total point of motion (MTPM) of the uncemented femoral component. Whiskers indicate 95% confidence interval

The greatest increase in mean MTPM (0.65 mm) occurred within the first 3 months. Afterwards, the curve flattened and stabilized, and the mean MTPM after 24 months was 0.96 mm (Fig. 4).

Mean MTPM was 0.84 mm (range 0.24–3.64 mm) after 6 months, 0.92 mm (range 0.17–4.93 mm) after 12 months and 0.96 mm (range 0.2–5.36 mm) after 24 months. Implant migration > 0.10 mm was observed between 12 and 24 months in 16% of patients (4 out of 25 patients).

**Fig. 5:**
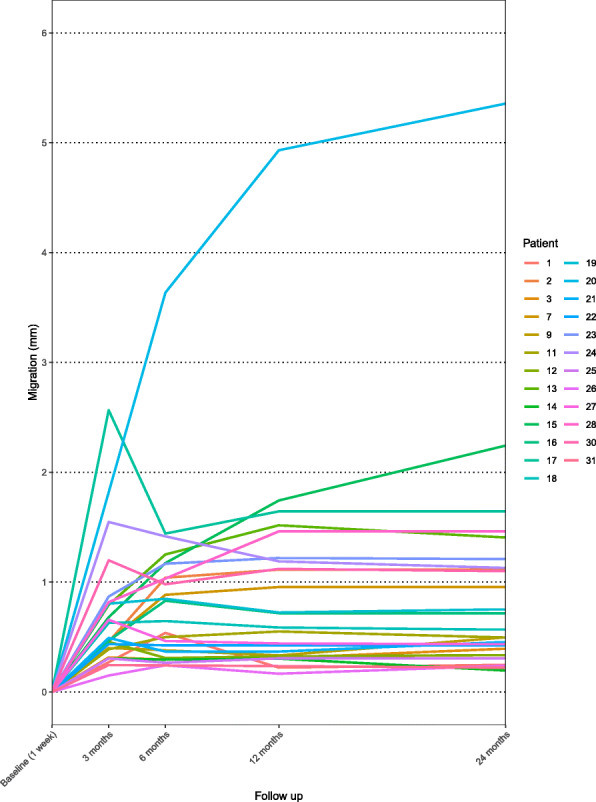
Maximal total point of motion (MTPM) of uncemented femoral component in individual patients

A spaghetti plot demonstrates the individual MTPM (Fig. 5). Patient number 20 initially had extremely high implant migration, which tended to stabilize after 12 months at 4.9 mm, and patient number 17 had high implant migration within the first 3 months, which stabilized after 6 months. Importantly, patient number 15 had implant migration that appeared to continue without stabilizing, as seen in the other patients. There have been no clinical complications observed so far.

**Fig. 6:**
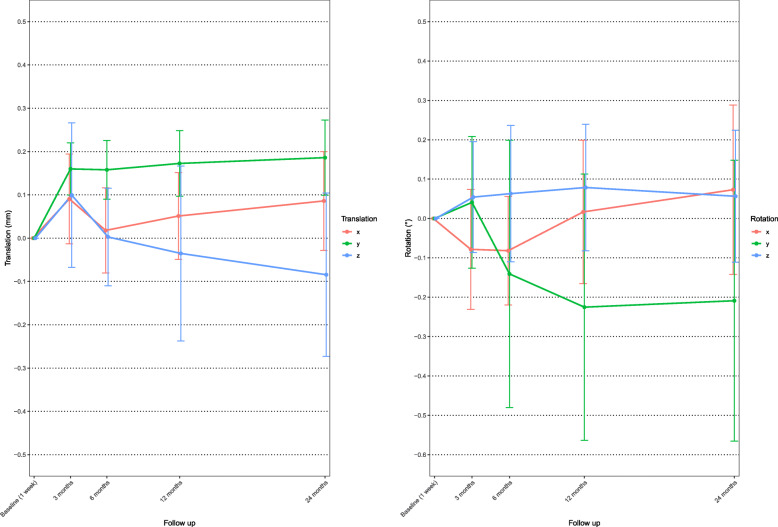
Mean X, Y, and Z rotation (right) and X, Y, and Z translation (left) of the uncemented femoral component. Whiskers indicate 95% confidence interval

The highest mean rotational and translational segmental motion was found around the Y axes (Fig. 6); mean rotation during the first 24 months was − 0.21°, where negative values indicate external rotation.

The mean CN was 58.4 (range 20.5–97.0) and mean ME was 0.16 (range 0.03–0.43). All CN values were acceptable, whereas one ME value (0.43) was above the maximum value of 0.35 as recommended by guidelines [24].

#### Tibia

**Fig. 7:**
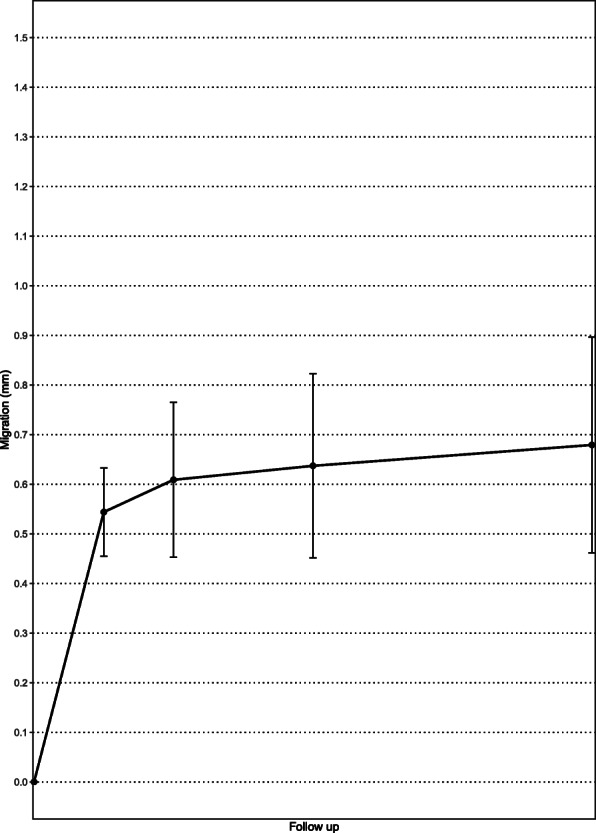
Mean maximal total point of motion (MTPM) of the cemented tibial component. Whiskers indicate 95% confidence interval

The precision error for measurement of MTPM from evaluation of the 22 double tibial examinations was 0.33 mm, 0.20°, 0.63°, and 0.21° for rotational segment motion, X, Y, and Z rotations, respectively, and 0.14 mm, 0.09 mm, and 0.19 mm, respectively, for the corresponding translational segment motion. The greatest increase in mean MTPM (0.54 mm) was seen after 3 months of follow up and then the curve considerably flattened as an expression of stabilization of the tibial component, with mean MTPM of 0.61 mm (range 0.17–1.99 mm) after 6 months, 0.65 mm (range 0.13–2.82 mm) after 12 months, and 0.69 mm (range 0.12–3.2 mm) after 24 months (Fig. 7). Implant migration greater than 0.2 mm was observed in 15% of patients (4 out of 27 patients) between 12 and 24 months. At 12 and 24 months of follow up there were 12 patients with MTPM ≤ 0.54 mm, 14 patients with MTPM between 0.54 and 1.6 mm, and 1 patient with MTPM > 1.6 mm (ME 0.29 and 0.32 at 12 and 24 months, respectively), which was therefore categorized as unacceptable.

**Fig. 8:**
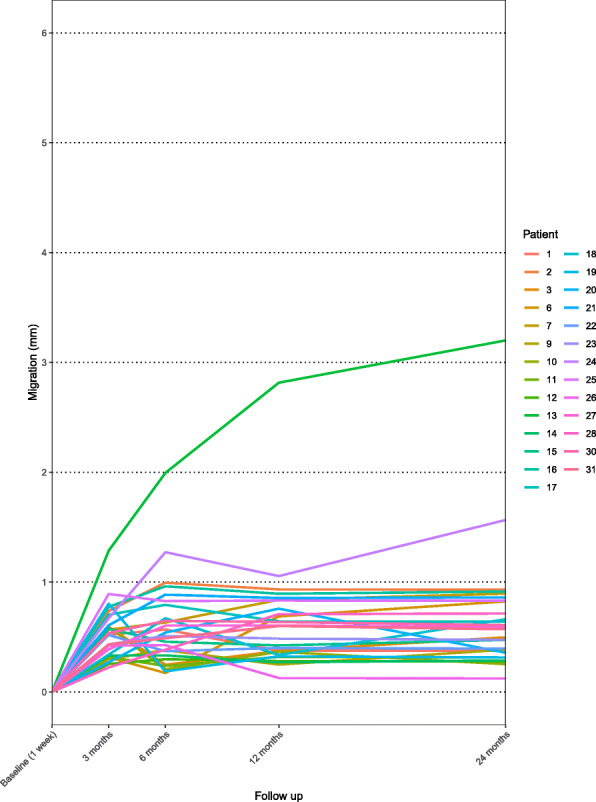
Maximal total point of motion (MTPM) of cemented tibial component in individual patients

The spaghetti plot for the tibial component showing the individual MTPM (Fig. 8) indicates high migration of 3.2 mm after 24 months in patient 13 and a late increase in migration (1.06 mm to 1.6 mm) between 12 and 24 months in patient 24. Migration appears not to have stabilized after 24 months in these two patients.

**Fig. 9:**
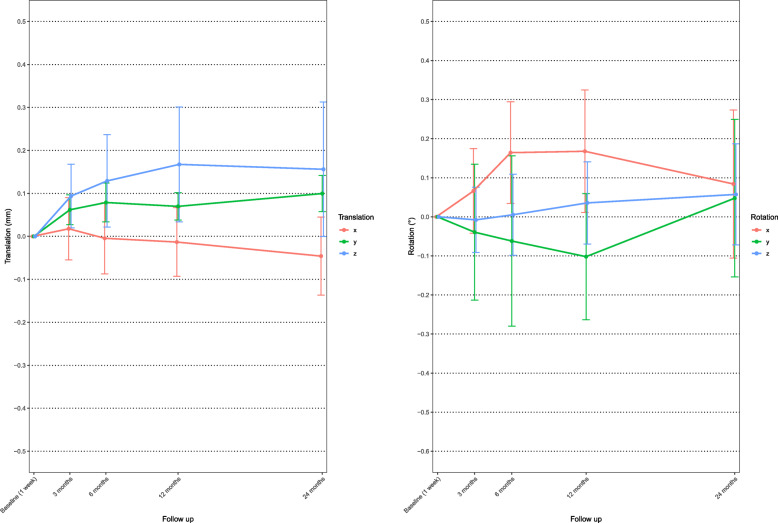
Mean X, Y, and Z rotation (right) and X, Y, and Z translation (left) of cemented tibial component. Whiskers indicate 95% confidence interval

Rotational and translational movement is reported in Fig. 9. The main movement responsible for MTPM at 3 months is rotation along the Y axes and at 6, 12, and 24 months it is translation along the Z axes.

Mean CN was 51.1 (range 32.9–133.1) and mean ME was 0.17 (range 0.06–0.4). All CN values were acceptable and one ME value (0.4) was above the maximum value of 0.35 as recommended by guidelines [24].

### DXA

The precision error expressed as the CV for measurement of BMD at each ROI was calculated from 16 double examinations. The CV for the distal femur was 1.4% (95CI 0.89–1.9), 1.3% (95CI 0.43-2.11), and 0.9% (95CI 0.5–1.4) for ROI I, ROI II, and ROI III, respectively. The corresponding results for the proximal tibia were 1.3% (95CI 0.69–1.95), 1.8% (95CI 0.86–2.68), and 2.1% (95CI 0.9–3.25), respectively.

**Fig. 10:**
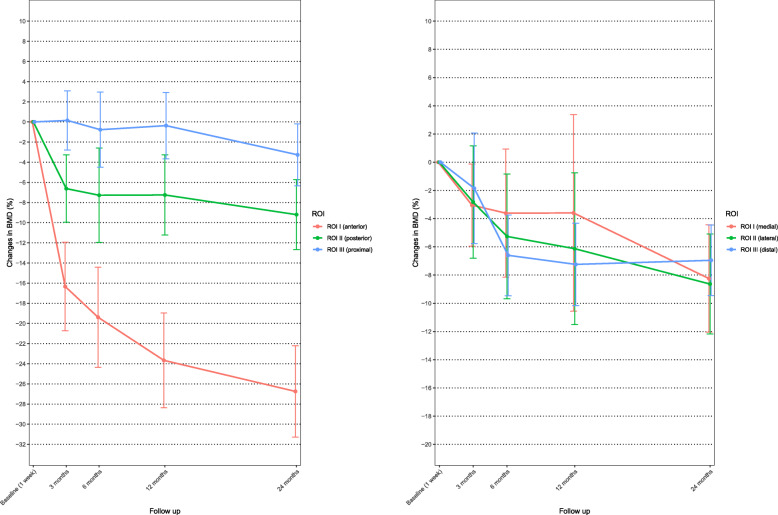
Mean bone mineral density (BMD) percentage change for region of interest (ROI) I, ROI II, and ROI III for the distal femur (left) and proximal tibia (right). Whiskers indicate 95% confidence interval

At both the distal femur and the proximal tibia and at all ROI, there was a statistically significant decrease in BMD at 2 years compared with the immediate postoperative measurement (Fig. 10).

**Fig. 11:**
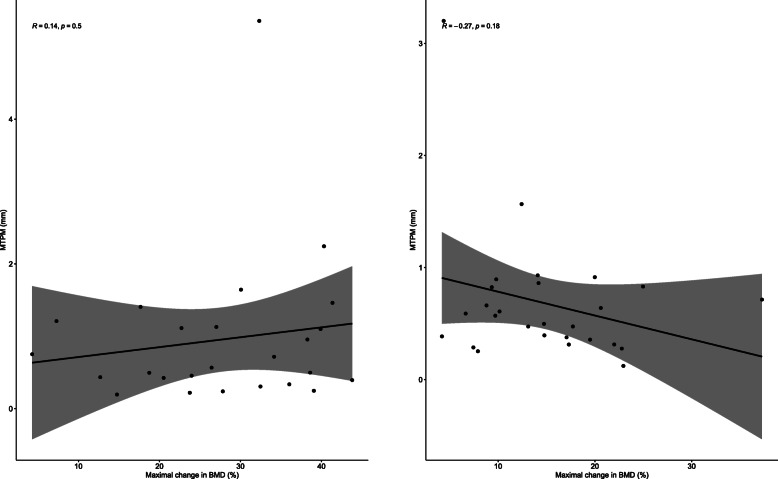
Analysis of correlation between maximal total point of motion (MTPM) and bone mineral density (BMD) 2 years after surgery. Femoral component (left) and tibial component (right). The gray area indicates 95% confidence interval

The greatest mean BMD decrease at the distal femur was at ROI I (anterior) with 26.7% decrease (95CI 17.3–36.1%) after 2 years, while the decrease at ROI II (posterior) and at ROI III (proximal) was 9.2% (95CI -3–21.5%) and 3.3% (95CI -5.55–12.1%) respectively. A decrease in BMD after 24 months was also observed in the proximal tibia and it was almost the same at all three ROI with 9.5% (95CI 4.7–14.3%) at ROI I (medial), 9.6% (95CI 2.5–16.7%) at ROI II (lateral), and 7.2% (95CI 0.6–13.8%) at ROI III (distal), respectively. There was no significant correlation between MTPM and BMD after 2 years (Fig. 11).

### Clinical results

The 2-year clinical outcome determined by the OKS (*n* = 29) showed a significant increase (*p* <0.001) from a score of 25 (range 13–38) preoperatively to 44 (range 35–48) at the 2-year follow up. The KSS for function increased from 54 (range 10–100) preoperatively to 94 (50–100) at 2 years (*p* <0.001), and the corresponding KSS clinical score increased from 38 (range 10–79) to 87 (range 60–90) (*p* <0.001).

## Discussion

A prospective follow up of 29 patients with uncemented femoral component and cemented asymmetrical tibial component was evaluated using MBRSA, DXA, and clinical outcome. We found that the uncemented femoral component had the highest MTPM within the first 3 months with mean migration of 0.65 mm and 16% of patients (4 out of 25 patients) with migration > 0.10 mm at 12–24 months.

Revisions related to femoral components, regardless of fixation, are rare [7, 27]. This may be one of the main reasons why the femoral component is less commonly evaluated with RSA compared to tibial components. A recent study suggests that annual migration < 0.09–0.10 mm is comparable with a good long-term outcome [27], but to our knowledge there have been no studies to estimate the proportion of implant migration and the risk of aseptic loosening with the femoral component.

Gao et al. [30] identified a median MTPM of 0.87 mm at 24 months postoperatively in younger patients (age < 60 years) and Nilsson et al. [31] reported a mean MTPM of 0.89 ± 0.08 mm.

The findings on femoral components in our study correspond well with previous studies [7, 30–32]. With a mean increase in MTPM < 0.10 mm per year we can expect a good long-term outcome. Four patients in our study had migration > 0.10 mm from 12 to 24 months; two of these had values fairly close to the proportion with 0.17 and 0.11 mm, but two outliers had very high migration (MTPM after 24 months 2.24 and 5.36 mm, respectively). With a rise in the proportion of 0.5 and 0.43 mm, correspondingly the ME was 0.3 and 0.12, respectively; these patients need to be followed further to evaluate their clinical outcome. No complications were observed at the 24-month follow up.

One possibility for further studies could be to examine the migration pattern of femoral components in patients who underwent revision due to aseptic loosening, to identify any pattern.

For the tibial components as with the femoral component, the greatest increase in mean MTPM (0.54 mm) was seen after 3 months of follow up. Mean MTPM was 0.61 mm after 6 months, 0.65 mm after 12 months, and 0.69 mm after 24 months. Between 12 and 24 months after surgery, 14.8% of patients (4 out of 27 patients) had migration > 0.2 mm.

Pijls et al. [28] identified association between early migration (MTPM at 12 months) and late implant revision (prosthesis survival after 5 years). A threshold of 0.54 mm MTPM after 1 year was categorized as an acceptable rate of aseptic loosing after 5 years, whereas the unacceptable threshold for MTPM was 1.6 mm, and values in between were considered components at risk [28]. In our study, 12 patients had MTPM ≤ 0.54 mm at 12 and 24 months of follow up, 14 patients had MTPM between 0.54 and 1.6 mm and were therefore (according to Pijls’ [28] classification) at risk of aseptic loosening after 5 years, and 1 patient had MTPM > 1.6 mm at 12 and 24 months (ME 0.29 and 0.32) follow up, which was therefore categorized as unacceptable. Importantly, note that no revision surgery was performed up to the 2-year follow up.

From Leande et al. [33] interpretation of the plot for cemented tibial components indicates 16 patients out of 58 patients at risk, with MTPM values > 0.54 at 1-year follow up, and 14 patients at risk at 2-year follow up, with 2 patients having MTPM values > 1.6, which is therefore considered unacceptable [33].

Many RSA studies have been effectuated using a different type of fixation and prosthesis design for the tibial component. If we compare our results with previous studies using cemented fixations [28, 29, 32–34], our results are similar or marginally higher. A 5-year follow up is already planned in this study and it is important to observe the components at risk.

Furthermore, Ryd et al. [6] state that MTPM migration > 0.2 mm from 1 to 2 years after surgery is a predictable factor for subsequent loosening of the components. In our study, 14.8% of patients (4 out of 27) had MTPM > 0.2 mm between 1 and 2 years, and therefore this should be considered when evaluating the prosthesis migration pattern in this study design.

A decrease in BMD of 26.7% was observed in ROI I at the distal femur after 24 months, and the respective decrease in ROI II and ROI III was 9.2% and 3.3%, respectively. The corresponding decrease in BMD at the proximal tibia in ROI I, II, and III was around 9%. The decrease in BMD at the distal femur and proximal tibia after TKA is a known consequence of postoperative adaptive bone remodeling [35–39]. BMD in the anterior part of the distal femur is clinically especially important in TKA because it is a common location for periprosthetic fractures [40–42]. Because BMD is closely related to trabecular bone strength [43], a significant decrease in BMD in this region will indicate an increased risk of periprosthetic fracture complications.

Quantitative studies have been performed on periprosthetic bone remodeling at the distal femur after primary TKA, but in general, the greatest bone loss is seen in the anterior part of the bone where the decrease in BMD typically reaches 23.6–36.0% after 2 years with uncemented femoral components [16, 35, 36]. Petersen et al. [18] identified a decrease in BMD of 44% in ROI I 1 year postoperatively.

The greatest decrease in BMD at the proximal tibia is often in ROI I (medial) and previous studies have identified a decrease with cemented tibial components between 4.4% after 1 year [35] and up to 38.6% after 2 years [44]. In our study, we identified a decrease in BMD of 9.5% in ROI I (medial) after 2 years, which is at the lower end of that found in previous studies [14, 16, 44–46]. The decrease in BMD in ROI II varies from 3% [35] to 20% [16] and in ROI III from 6.5% [35] to 36.8% [44]. In our study decreases in BMD of 9.6% in ROI II and 7.2% in ROI III were observed and this corresponds well with the findings of previous studies [14, 15, 45, 46].

To our knowledge, there are no studies to indicate the range of decrease in BMD associated with periprosthetic fracture; one of the reasons for this could be that periprosthetic fracture is not only associated with a decrease in BMD but also has a multifactorial genesis. Decrease in BMD in the present study was caused by local adaptive bone remodeling.

### Limitations

This study has no randomization between a current standard prosthesis and the new implant, which would be the preferred way to test a new implant; with the patients blinded to the type of prosthesis, the clinical outcome could be determined more accurately. Results from 29 patients for one type of prosthesis are acceptable for studying implant migration and adaptive bone remodeling after TKA, but to interpret functional results more patients are needed.

## Conclusion

Migration patterns for femoral component and changes in BMD in our study correspond well with findings in previous studies, and we observed marginally higher migration with the tibial component. There was no significant correlation between MTPM and BMD. Those components at risk need further evaluation with 5-year postoperative follow up.

## Supplementary Information

**Additional file 1.**

## Data Availability

The dataset used and/or analyzed during the current study are available from the corresponding author on reasonable request.

## Abbreviations

95CI: 95% Confidence interval
BMD: Bone mineral density
CAD: Computer-aided design
CN: Condition number
DXA: Dual-energy x ray absorptiometry
KSS: Knee Society score
MBRSA: Model-based radiostereometric analysis
ME: Mean error
MTPM: Maximal total point of motion
OA: Osteoarthritis
OKS: Oxford knee score
PACS: Picture archiving and communication system
ROI: Region(s) of interest
RSA: Radiostereometric analysis
TKA: Total knee arthroplasty
