# Supplementary material for: Bone remodeling and implant migration of uncemented femoral and cemented asymmetrical tibial components in total knee arthroplasty - DXA and RSA evaluation with 2-year follow up
Source: Knee Surg Relat Res. 2021 Aug 17;33:25. doi: 10.1186/s43019-021-00111-5 (PMC8369662; doi:10.1186/s43019-021-00111-5)
Supplement: Supplementary file 1 — Additional file 1. [file 43019_2021_111_MOESM1_ESM.docx]

**Supplementary**

|  |  | **Mean** | **Range** | **SD** | **95CI** |
| --- | --- | --- | --- | --- | --- |
| **Mean MTPM (mm)** | 3 months | 0.65 | 0.15 – 2.57 | 0.52 | 0.44 – 0.87 |
|  | 6 months | 0.84 | 0.24 – 3.64 | 0.72 | 0.53 – 1.16 |
|  | 12 months | 0.92 | 0.17 – 4.93 | 0.97 | 0.52 – 1.31 |
|  | 24 months | 0.96 | 0.2 – 5.36 | 1.06 | 0.52 – 1.39 |
| **X-rotation (°)** | 3 months | -0.08 | -1.25 – 0.53 | 0.38 | -0.24 – 0.087 |
|  | 6 months | -0.08 | -0.74 – 0.6 | 0.35 | -0.23 – 0.068 |
|  | 12 months | 0.02 | -0.76 – 1.11 | 0.47 | -0.18 – 0.21 |
|  | 24 months | 0.07 | -0.83 – 1.41 | 0.56 | -0.16 – 0.3 |
| **Y-rotation (°)** | 3 months | 0.041 | -0.7 – 1.37 | 0.42 | -0.14 – 0.22 |
|  | 6 months | -0.14 | -3.46 – 0.93 | 0.85 | -0.51 – 0.23 |
|  | 12 months | -0.23 | -4.09 – 0.65 | 0.88 | -0.59 – 0.14 |
|  | 24 months | -0.21 | -4.11 – 0.92 | 0.93 | -0.59 – 0.17 |
| **Z-rotation (°)** | 3 months | 0.054 | -0.71 – 0.91 | 0.35 | -0.098 – 0.21 |
|  | 6 months | 0.063 | -0.72 – 1.17 | 0.43 | -0.12 – 0.25 |
|  | 12 months | 0.079 | -0.64 – 1.14 | 0.42 | -0.09 – 0.25 |
|  | 24 months | 0.056 | -0.66 – 0.96 | 0.44 | -0.12 – 0.24 |
| **X-translation (mm)** | 3 months | 0.09 | -0.37 – 0.9 | 0.26 | -0.02 – 0.2 |
|  | 6 months | 0.02 | -0.65 – 0.57 | 0.25 | -0.09 – 0.12 |
|  | 12 months | 0.05 | -0.43 – 0.96 | 0.26 | -0.06 – 0.16 |
|  | 24 months | 0.09 | -0.41 – 0.89 | 0.3 | -0.04 – 0.21 |
| **Y-translation (mm)** | 3 months | 0.16 | -0.04 – 0.67 | 0.15 | 0.09 – 0.22 |
|  | 6 months | 0.16 | -0.4 – 0.68 | 0.17 | 0.08 – 0.23 |
|  | 12 months | 0.17 | -0.06 – 0.81 | 0.2 | 0.09 – 0.25 |
|  | 24 months | 0.19 | -0.04 – 1.02 | 0.23 | -0.12 – 0.12 |
| **Z-translation (mm)** | 3 months | 0.1 | -0.35 – 1.71 | 0.43 | 0.09 – 0.28 |
|  | 6 months | 0.002 | -0.88 – 0.47 | 0.28 | -0.08 – 0.28 |
|  | 12 months | -0.04 | -1.75 – 1.45 | 0.53 | -0.25 – 0.18 |
|  | 24 months | -0.08 | -2.05 – 0.47 | 0.49 | -0.29 – 0.19 |

Femoral component migration, rotation and translation measured with MBRSA.

|  |  | **Mean** | **Range** | **SD** | **95CI** |
| --- | --- | --- | --- | --- | --- |
| **Mean MTPM (mm)** | 3 months | 0.54 | 0.22 – 1.29 | 0.24 | 0.45 – 0.64 |
|  | 6 months | 0.61 | 0.17 – 1.99 | 0.41 | 0.44 – 0.78 |
|  | 12 months | 0.65 | 0.13 – 2.82 | 0.5 | 0.44 – 0.84 |
|  | 24 months | 0.69 | 0.12 – 3.2 | 0.59 | 0.45 – 0.91 |
| **X-rotation (°)** | 3 months | 0.066 | -0.71 – 0.69 | 0.29 | -0.05 – 0.18 |
|  | 6 months | 0.16 | -0.23 – 1.37 | 0.34 | 0.024 – 0.3 |
|  | 12 months | 0.17 | -0.22 – 1.96 | 0.42 | 0.00023 – 0.33 |
|  | 24 months | 0.084 | -0.54 – 2.27 | 0.51 | -0.12 – 0.29 |
| **Y-rotation (°)** | 3 months | -0.39 | -0.92 – 0.88 | 0.46 | -0.23 – 0.19 |
|  | 6 months | -0.06 | -1.28 – 1.04 | 0.57 | -0.3 – 0.17 |
|  | 12 months | -0.10 | -1.32 – 0.7 | 0.44 | -0.27 – 0.07 |
|  | 24 months | 0.048 | -1.37 – 1 | 0.54 | -17 – 0.26 |
| **Z-rotation (°)** | 3 months | -0.008 | -0.59 – 0.43 | 0.22 | -0.097 – 0.084 |
|  | 6 months | 0.005 | -0.71 – 0.6 | 0.27 | -0.11 – 0.12 |
|  | 12 months | 0.036 | -0.83 – 0.65 | 0.0.28 | -0.077 – 0.15 |
|  | 24 months | 0.057 | -0.84 – 0.71 | 0.35 | -0.081 – 0.195 |
| **X-translation (mm)** | 3 months | 0.018 | -0.34 – 0.47 | 0.19 | -0.06 – 0.01 |
|  | 6 months | -0.0045 | -0.39 – 0.59 | 0.22 | -0.93 – 0.08 |
|  | 12 months | -0.013 | -0.36 – 0.69 | 0.22 | -0.099 – 0.072 |
|  | 24 months | -0.046 | -0.46 – 0.71 | 0.25 | -0.14 – 0.05 |
| **Y-translation (mm)** | 3 months | 0.06 | -0.13 – 0.26 | 0.09 | 0.024 – 0.1 |
|  | 6 months | 0.079 | -0.16 – 0.39 | 0.12 | 0.03 – 0.127 |
|  | 12 months | 0.07 | -0.14 – 0.24 | 0.09 | 0.036 – 0.104 |
|  | 24 months | 0.1 | -0.18 – 0.35 | 0.11 | 0.054 – 0.14 |
| **Z-translation (mm)** | 3 months | 0.09 | -0.31 – 0.68 | 0.2 | 0.015 – 0.17 |
|  | 6 months | 0.13 | -0.3 – 1.2 | 0.28 | 0.014 – 0.24 |
|  | 12 months | 0.17 | -0.19 – 1.8 | 0.36 | 0.025 – 0.31 |
|  | 24 months | 0.16 | -0.5 – 2.06 | 0.42 | -0.011 – 0.32 |

Tibial component migration, rotation and translation measured with MBRSA.

|  | | | **1 week** | **3 months** | **6 months** | **12 months** | **24 months** | **0-24 months t-test**  **p-value** |
| --- | --- | --- | --- | --- | --- | --- | --- | --- |
| **Femur** | ROI I  (anterior) | Mean | 1.112 | 0.928 | 0.896 | 0.851 | 0.815 | <0.001 |
|  |  | Range | (0.664,1.902) | (0.529,1.528) | (0.471,1.427) | (0.425,1.419) | (0.407,1.399) |  |
|  |  | △BMD% |  | -16.3 | -19.4 | -23.7 | -26.7 |  |
|  |  | 95CI |  | (-20.7,-11.8) | (-24.4,-14.4) | (-28.4,-19.0) | (-31.3,-22.2) |  |
|  | ROI II  (posterior) | Mean | 1.496 | 1.391 | 1.387 | 1.385 | 1.355 | <0.001 |
|  |  | Range | (0.951,2.209) | (0.947,2.064) | (0.639,2.172) | (0.892,2.142) | (0.874,2.123) |  |
|  |  | △BMD% |  | -6.6 | -7.3 | -7.2 | -9.2 |  |
|  |  | 95CI |  | (-10.0,-3.3) | (-12.0,-2.6) | (-11.2,-3.3) | (-12.7,-5.7) |  |
|  | ROI III  (proximal) | Mean | 1.112 | 1.105 | 1.097 | 1.101 | 1.070 | 0.02 |
|  |  | Range | (0.64,1.66) | (0.74,1.6) | (0.63,1.6) | (0.63,1.64) | (0.62,1.58) |  |
|  |  | △BMD% |  | 0.15 | -0.77 | -0.37 | -3.3 |  |
|  |  | 95CI |  | (-2.8,3.1) | (-4.5,3.0) | (-3.7,2.9) | (-6.3, 0.2) |  |
| **Tibia** | ROI I  (medial) | Mean | 0.989 | 0.953 | 0.942 | 0.934 | 0.896 | <0.001 |
|  |  | Range | (0.640,1.462) | (0.718,1.370) | (0.722,1.342) | (0.683,1.276) | (0.680,1.145) |  |
|  |  | △BMD% |  | -3.05 | -3.6 | -3.6 | -8.2 |  |
|  |  | 95CI |  | (-6.0,0.1) | (-8.1,0.9) | (-10.6,3.4) | (-12.1,-4.4) |  |
|  | ROI II  (lateral) | Mean | 1.027 | 0.991 | 0.961 | 0.949 | 0.928 | <0.001 |
|  |  | Range | (0.753,1.413) | (0.757,1.392) | (0.710,1.379) | (0.686,1.375) | (0.739,1.327) |  |
|  |  | △BMD% |  | -2.8 | -5.3 | -6.2 | -8.6 |  |
|  |  | 95CI |  | (-6.8,1.2) | (-9.7,-0.8) | (-11.5,-0.7) | (-12.2,-5.1) |  |
|  | ROI III  (distal) | Mean | 1.133 | 1.111 | 1.057 | 1.050 | 1.052 | <0.001 |
|  |  | Range | (0.823,1.445) | (0.772,1.465) | (0.736,1.435) | (0.750,1.483) | (0.725,1.425) |  |
|  |  | △BMD% |  | -1.9 | -6.6 | -7.2 | -7.0 |  |
|  |  | 95CI |  | (-4.9,1.2) | (-9.5,-3.7) | (-10.2,-4.3) | (-9.5-4.4) |  |

BMD measured with DXA for the distal femur and proximal tibia.
